# Metabolic Disorders in Patients with Chronic Hepatitis B Virus Infection: Coffee as a Panacea? (ANRS CO22 Hepather Cohort)

**DOI:** 10.3390/antiox11020379

**Published:** 2022-02-14

**Authors:** Tangui Barré, Hélène Fontaine, Stanislas Pol, Clémence Ramier, Vincent Di Beo, Camelia Protopopescu, Fabienne Marcellin, Morgane Bureau, Marc Bourlière, Céline Dorival, Ventzislava Petrov-Sanchez, Tarik Asselah, Elisabeth Delarocque-Astagneau, Dominique Larrey, Jean-Charles Duclos-Vallée, Fabrice Carrat, Patrizia Carrieri, on behalf of the ANRS/AFEF Hepather Study Group

**Affiliations:** 1 Aix Marseille Univ. Inserm, IRD, SESSTIM, Sciences Economiques & Sociales de la Santé & Traitement de l’Information Médicale, ISSPAM, 13005 Marseille, France; tangui.barre@inserm.fr (T.B.); clemence.ramier@inserm.fr (C.R.); vincent.di-beo@inserm.fr (V.D.B.); camelia.protopopescu@inserm.fr (C.P.); fabienne.marcellin@inserm.fr (F.M.); morgane-diane.bureau@inserm.fr (M.B.); mbourliere@hopital-saint-joseph.fr (M.B.); 2 Université de Paris, AP-HP, Hôpital Cochin, Département d’Hépatologie/Addictologie, 75014 Paris, France; helene.fontaine@aphp.fr (H.F.); stanislas.pol@aphp.fr (S.P.); 3 Hôpital St. Joseph, Service d’Hépato-Gastroentérologie, 13008 Marseille, France; 4 Institut National de la Santé et de la Recherche Médicale (INSERM), Institut Pierre Louis d’Epidémiologie et de Santé Publique, Sorbonne Université, 75646 Paris, France; celine.dorival@iplesp.upmc.fr; 5 ANRS MIE (France Recherche Nord & Sud Sida-HIV Hépatites|Maladies Infectieuses Emergentes), Unit for Basic and Clinical Research on Viral Hepatitis, 73013 Paris, France; ventzislava.petrov-sanchez@anrs.fr; 6 Université de Paris, Centre de Recherche sur L’inflammation, INSERM UMR1149, 75018 Paris, France; tarik.asselah@aphp.fr; 7 Department of Hepatology, AP-HP, Hôpital Beaujon, 92110 Clichy, France; 8 Université Paris-Saclay, UVSQ, Inserm, CESP, Team Anti-Infective Evasion and Pharmacoepidemiology, 78180 Montigny, France; elisabeth.delarocque-astagneau@uvsq.fr; 9 AP-HP, GHU Paris Saclay University, Raymond Poincaré Hospital, Epidemiology and Public Health Department, 92380 Garches, France; 10 Liver Unit-IRB-INSERM 1183, Hôpital Saint Eloi, 34090 Montpellier, France; dom-larrey@chu-montpellier.fr; 11 AP-HP Hôpital Paul-Brousse, Centre Hépato-Biliaire, Villejuif, UMR-S 1193, Université Paris-Saclay, FHU HEPATINOV, 94800 Villejuif, France; jean-charles.duclos-vallee@aphp.fr; 12 Hôpital Saint-Antoine, Unité de Santé Publique, Assistance Publique-Hôpitaux de Paris (AP-HP), 75012 Paris, France; fabrice.carrat@iplesp.upmc.fr

**Keywords:** coffee, tea, hepatitis B, metabolic syndrome, dyslipidemia, hypertension, polyphenol

## Abstract

People living with chronic hepatitis B virus (HBV) infection are at high risk of liver disease progression, which is positively associated with metabolic disorders, but inversely associated with dyslipidemia. Diet, including dietary antioxidants, is a lever of metabolic disorder management. In particular, elevated coffee consumption is associated with different metabolic outcomes in the general population. We aimed to test whether such associations occur in HBV-infected people. Based on cross-sectional data from the ANRS CO22 Hepather cohort, we performed logistic regression models with (i) dyslipidemia, (ii) hypertension, and (iii) diabetes as outcomes, and with demographic, clinical, and socio-behavioral (including coffee consumption) data as explanatory variables. Among 4746 HBV-infected patients, drinking ≥3 cups of coffee per day was associated with a higher risk of dyslipidemia (adjusted odds ratio [95% confidence interval] 1.49 [1.10–2.00], *p* = 0.009) and a lower risk of hypertension (0.64 [0.50–0.82], *p* = 0.001). It was not associated with diabetes. Elevated coffee consumption was associated with a higher risk of dyslipidemia and a lower risk of hypertension in HBV-infected patients, two effects expected to be associated with favorable clinical outcomes. Further studies should test whether such metabolic benefits translate into reduced mortality risk in this population.

## 1. Introduction

Hepatitis B virus (HBV) infection affects hosts’ metabolic pathways. It is associated with lower levels of serum total cholesterol, high-density lipoprotein (HDL) cholesterol, and triglycerides (TG) [1,2]. Metabolic syndrome (MetS) is defined as having three or more of the following cardiovascular risk factors: central obesity, elevated TG, reduced HDL-cholesterol, elevated blood pressure, and elevated fasting glucose [3]. Accordingly, people infected with HBV have a lower prevalence of MetS [4,5]. More specifically, a strong inverse association was found between HBV infection and elevated TG, as well as a slight inverse association with both reduced HDL-cholesterol and elevated fasting glucose. No association was found with central obesity or elevated blood pressure [4].

However, MetS is itself a risk factor for hepatocellular carcinoma (HCC), non-HCC cancer, liver-related and all-cause mortality [5,6,7] in people infected with HBV, who are over 10 times more likely to die from liver cancer than people not infected [8]. Therefore, the relatively low prevalence of MetS in this population should not divert attention from its potential lethal effects.

The five MetS components do not appear to play the same role in the pathogenesis of liver disease in HBV-infected persons [5]. For example, total cholesterol was significantly lower in HBV patients who had advanced chronic liver disease than in those who did not [9]. Furthermore, hypercholesterolemia in HBV patients was independently associated with decreased liver cancer mortality, irrespective of statin use [10]. Moreover, low levels of triglycerides were associated with HCC in HBV-infected patients with type 2 diabetes [11,12]. Accordingly, low levels of cholesterol and triglycerides may be risk factors for liver disease in this population. 

Modulating diet is one of the main strategies available for the prevention and treatment of MetS [13]. For instance, the Dietary Approaches to Stop Hypertension (DASH) diet is recognized as an effective dietary intervention to reduce blood pressure [14]. Furthermore, adherence to the Mediterranean diet was associated with lower TG and higher HDL-cholesterol [15]. These two dietary patterns are characterized by high amounts of antioxidants, which probably play a role in the observed protective effects [16,17]. While data are still inconclusive, dietary antioxidants may constitute protective factors against liver disease in HBV-infected patients [18,19,20]. Polyphenols comprise a large family of molecules bearing one or more phenolic rings. Most possess antioxidant (and anti-inflammatory) properties [21]. Coffee and tea constitute two main food sources of polyphenols [22,23,24]. In the general population, coffee consumption is associated with higher total cholesterol levels. Results for TG levels are less consistent [25,26,27]. Conversely, tea consumption seems to lower total cholesterol; again, results for TG levels are inconsistent [26,28]. With regard to hypertension, a blood pressure-lowering effect has been consistently documented for both beverages [29,30,31]. Moreover, coffee consumption seems to be more robustly associated with beneficial diabetes-related outcomes than tea [32,33,34]. To our knowledge, none of the above associations have been explored in people chronically infected with HBV. Managing lipid serum levels and blood pressure in HBV-infected patients—including through diet modulation—may be a lever to reduce the elevated risk of liver disease progression and HCC in this population for whom functional cure remains rare [8,35,36]. Accordingly, the present study aimed to test whether coffee consumption is associated with dyslipidemia, hypertension, and diabetes in HBV-infected patients.

## 2. Materials and Methods

### 2.1. Design and Participants

ANRS CO22 Hepather is a French, national, multicenter, prospective, observational cohort study of patients with hepatitis B or C virus infection (ClinicalTrials.gov registration number NCT01953458). It is promoted by the ANRS Emerging Infectious Diseases. Initiated in 2012, Hepather aims to increase knowledge of viral hepatitis [37]. Thirty-two hospital clinical centers, spread over French territory, are involved in data collection. 

Sociodemographic, clinical, and biological data were collected at the cohort enrolment visit. Patients are followed on a yearly basis, and supplemental data are collected during visits related to particular events (e.g., HBV or hepatitis C-HCV-therapy initiation). Written informed consent was obtained from each patient before enrolment. The protocol was developed in accordance with the Declaration of Helsinki and French law for biomedical research, and was approved by the CPP Ile de France 3 ethics committee (Paris, France) and the French Regulatory Authority (ANSM).

### 2.2. Study Population

The main exclusion criterion in Hepather was HIV coinfection. For the present study, the study population comprised patients with chronic HBV virus infection at cohort enrolment (defined by positive HBsAg for at least six months). Patients with HCV or HDV coinfection were excluded from the study population, as were patients with no data on coffee consumption and those with unavailable data for the three outcomes (i.e., dyslipidemia, hypertension, and diabetes).

### 2.3. Data Collection

We used data collected during the cohort enrolment visit, where physicians completed a structured questionnaire during a face-to-face interview with their patients, and collected anthropometric measurements, as well as blood and urine samples.

The questionnaire collected clinical and sociodemographic data including gender, age, country of birth, time since HBV diagnosis, cannabis use, tobacco use, current and past alcohol use (number of standard drinks per day), current coffee consumption (number of cups per day), current tea consumption (number of cups per day), living with a partner (yes/no), average monthly household income, and educational level. Body height, weight, and waist circumference were measured. Data derived from enrolment blood samples included platelet count (platelets/L), aspartate aminotransferase (AST, IU/L), and alanine aminotransferase (ALT, IU/L) levels, and gamma glutamyltransferase (GGT, IU/L) level.

Information concerning the existence of medical comorbidities (and associated treatments) at enrolment, including on diabetes, hypertension, hypercholesterolemia and hypertriglyceridemia, was also gathered during the inclusion visit using an electronic case report form.

### 2.4. Outcomes

Three outcomes were selected for this study. The first, dyslipidemia, was defined as the presence of hypercholesterolemia or hypertriglyceridemia, or receiving treatments for this type of disorder. The second, hypertension, was defined as the presence of hypertension or receiving treatment for it. Finally, the third outcome, diabetes, was defined as the diagnosis of diabetes or associated treatment receipt.

### 2.5. Explanatory Variables

For coffee consumption, a three-category variable was created based on three thresholds: zero, one, and three or more cups per day. The one cup per day threshold was used to test for a potential dose response relationship between coffee consumption and the three study outcomes. The three cups or more per day threshold was chosen based on previous results showing the potential protective effect of this level of consumption on liver stiffness and mortality in patients likely to develop liver disease [38,39]. Given the expected dose-response effect of coffee consumption [29,40], we also tested the higher threshold of four cups per day when the three cups per day threshold was not significantly associated with the outcome.

For tea consumption, as the dose response is less clearly documented, two alternative thresholds were considered to characterize elevated tea consumption: one cup per day (i.e., daily consumption), and three or more cups per day. This led to two dichotomized variables.

Alcohol consumption was classified into four categories based on the threshold for unhealthy alcohol use (defined as >2 standard drinks per day for women and >3 standard drinks per day for men, in accordance with the French National Authority for Health [41]) as follows: abstinent (i) with or (ii) without a history of unhealthy alcohol use, (iii) current unhealthy alcohol use, and (iv) current moderate alcohol use (i.e., non-abstinent and healthy use). For both cannabis and tobacco use, participants were classified in the ‘current’, ‘former’, or ‘never’ user categories.

Poverty was defined as a standard of living lower than the 2015 French poverty threshold (1015 euros/month) [42]. Standard of living was calculated as disposable income divided by the number of consumption units in the household. Educational level was dichotomized into having an upper secondary school certificate or not.

Liver fibrosis was assessed using the FIB-4 index, which is a noninvasive marker of fibrosis calculated using age, AST level, ALT level, and platelet count, with the following formula: (age [years] × AST [IU/L])/(platelet count [109/L] × ALT [IU/L])1/2. The presence of advanced fibrosis was defined as an FIB-4 index > 3.25 [43,44]. 

### 2.6. Statistical Analyses

Study sample characteristics were compared between participants according to their coffee consumption (zero, one, and three or more cups per day). Chi-square and Kruskal–Wallis tests were used for categorical and continuous variables, respectively. Study sample characteristics were also compared between included patients and those patients excluded from the study population because of missing data.

Three separate logistic regression models were performed to test for an association between the use of the two studied polyphenol-rich beverages (i.e., coffee and tea) and the three study outcomes at cohort enrolment, after adjustment for other potential predictors. Associations were assessed by (adjusted) odds ratios ((a)ORs). Only variables with a liberal *p*-value < 0.20 in the univariable analyses were considered eligible for the multivariable model. The final multivariable model was built using a backward stepwise procedure, and the likelihood ratio test (*p* < 0.05) was used to define the variables to maintain in the final model.

For each outcome (i.e., dyslipidemia, hypertension, and diabetes), three multivariable models were run to better characterize the associations between the consumption variables and the outcome. In the first model (model 1), advanced liver fibrosis (FIB-4 index > 3.25) and the two metabolic disorders (i.e., outcomes) other than the outcome in question were not considered eligible as explanatory variables, as advanced liver fibrosis may directly cause diabetes [45,46], and metabolic disorders are likely to be co-occurring and to share a common pathogenesis pathway. In the second (model 2) and third (model 3) models, advanced liver fibrosis and metabolic disorders, respectively, were tested as potential explanatory variables.

To test for a dose response effect of coffee and tea, we also performed an alternative model to model 1 for each outcome, with hot beverage consumption considered as a continuous variable (i.e., cups per day). The other parameters were similar to those in model 1.

All analyses were performed with Stata software version 17.0 for Windows (StataCorp LP, College Station, TX, USA).

## 3. Results

### 3.1. Study Population Characteristics

**Figure 1:**
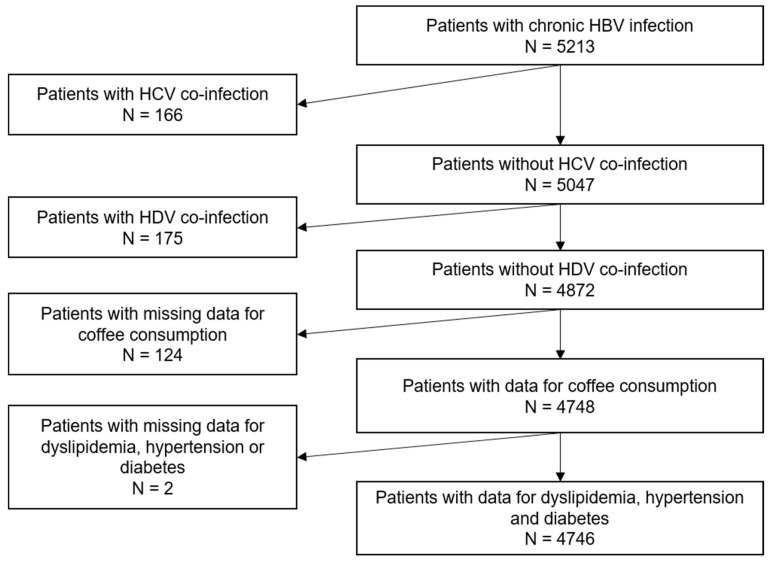
Flow chart of the study population (ANRS CO22 Hepather cohort). HBV, hepatitis B virus; HCV, hepatitis C virus; HDV, hepatitis D virus.

The study population comprised 4746 participants (Figure 1).

**Figure 2:**
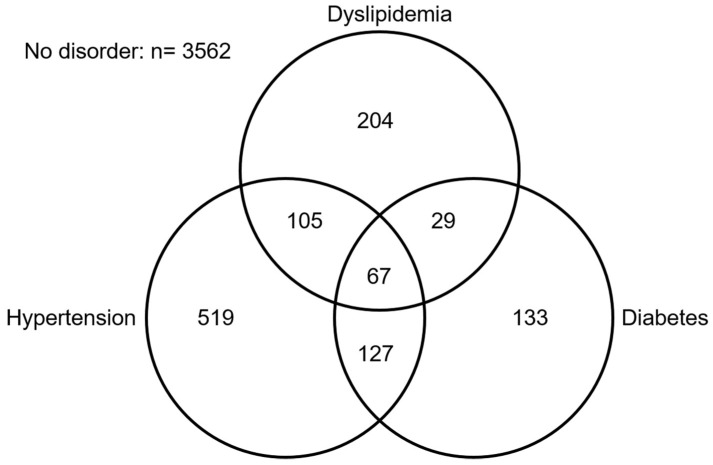
Frequencies of metabolic disorders in the study population (ANRS CO22 Hepather cohort).

Participants’ characteristics according to their coffee consumption level are provided in Table 1. Most were men (63.3%), and the median age was 43 years (interquartile range [34,35,36,37,38,39,40,41,42,43,44,45,46,47,48,49,50,51,52,53,54]). Just over a quarter (26.8%) had elevated coffee consumption, while 39.1% had daily tea consumption. A quarter (24.9%) had at least one metabolic disorder, the most prevalent being hypertension (Figure 2). Characteristics of included vs. excluded participants are given in Supplementary Table S1.

### 3.2. Factors Associated with Dyslipidemia

The results of univariable and multivariable logistic regressions for dyslipidemia are given in Table 2. Coffee consumption was associated with the outcome in all three multivariable models. Current elevated coffee consumption was associated with a higher likelihood of dyslipidemia in all three models (adjusted odds ratio (aOR) between 1.49 and 1.62), while moderate consumption only approached statistical significance. Other risk factors identified in all three models were older age and former or current tobacco use (Table 2). Daily tea consumption was associated with a lower likelihood of dyslipidemia in univariable analysis only.

When coffee and tea consumption were considered continuous variables, they were, respectively, positively (aOR 1.10, *p* < 0.001) and negatively (aOR 0.92, *p* = 0.029) associated with the outcome in univariable analyses. No such association was observed in the multivariable analysis (data not shown).

### 3.3. Factors Associated with Hypertension

The results of univariable and multivariable logistic regressions for hypertension are given in Table 3. Coffee consumption was associated with the outcome in all three multivariable models. Current elevated coffee consumption was associated with a lower likelihood of hypertension in all three models (adjusted odds ratio (aOR) between 0.64 and 0.66), while moderate consumption was not. Other risk factors identified in all three models were older age, being born in Sub-Saharan Africa, overweight, obesity, and current tobacco use (Table 3). Daily tea consumption was associated with a lower likelihood of hypertension in univariable analysis only.

When coffee and tea consumption were considered as continuous variables, they were both negatively associated with the outcome in univariable analyses, but only coffee consumption remained in multivariable analysis (aOR 0.90 [95% confidence interval] [0.85; 0.95], *p* < 0.001) (data not shown).

### 3.4. Factors Associated with Diabetes

The results of univariable and multivariable logistic regressions for diabetes are given in Table 4. Coffee consumption was not associated with diabetes in multivariable analyses. Neither was it associated with the outcome when the threshold for elevated consumption was raised to four cups per day. Risk factors identified in all three multivariable models were older age, overweight and obesity, and living in poverty. Tea consumption was not associated with diabetes (Table 4).

When considered as continuous variables, neither coffee nor tea consumption was associated with the outcome in univariable analyses (data not shown).

## 4. Discussion

In a large cohort of patients chronically infected with HBV, we found that elevated coffee consumption (≥3 cups per day) was associated with a higher risk of dyslipidemia, a lower risk of hypertension, and was not associated with diabetes. Tea consumption was not associated with any of these three outcomes.

These results were independent of BMI status, and remained valid after adjustment for advanced liver fibrosis and co-occurring metabolic disorders. FIB-4 may not be accurate in participants currently treated for HBV infection. Accordingly, results from model 2 should be cautiously interpreted. The dose response relationship that we observed for hypertension reflects the findings from previous studies [29,40,48]. The observed effects of coffee consumption on hypertension and dyslipidemia may partly mediate the hepatic benefits of coffee consumption previously highlighted in the HBV-infected population [49,50].

Our results for dyslipidemia and hypertension are consistent with those found in the general population [25,26,27,29,51]. In the general population, significant positive nonlinear associations between coffee consumption and the increase in total cholesterol, low-density lipoprotein cholesterol, and TG levels have been suggested, and coffee consumption may be associated with an elevated risk of dyslipidemia and cardiovascular diseases [51]. However, the anti-dyslipidemic properties of coffee consumption have also been documented, as have several putative-related mechanisms [52,53]. The possible lipid-raising effect of coffee might be partly imputable to cafestol and kahweol, two natural diterpenes that seem to have a modulatory effect on the low-density lipoprotein receptor [54,55], and may affect regulatory enzymes in the bile acid synthesis process and plasma lipid transfer protein levels [54]. Discrepancies between studies could be explained by an existing hypothesis that there is a differential lipid-raising effect between filtered and unfiltered coffee, the former having a weaker effect thanks to the filter’s capacity to retain cafestol. However, studies to date have provided no definitive evidence for such a differential effect [56,57,58]. 

In terms of HBV-infected patients, a population prone to liver cancer, this possible lipid-raising effect is of particular interest. HBV infection is associated with lower levels of serum total cholesterol, HDL cholesterol, and TG [1,2]. Furthermore, lower serum cholesterol is associated with more advanced stages of liver disease in this population [9,59], while higher serum cholesterol is associated with better liver cancer survival [10]. This inverse relationship between serum cholesterol and liver cancer has also been found in the general population [60]. Therefore, elevated levels of cholesterol and/or TG may be associated with better clinical outcomes in HBV-infected patients.

Just as for dyslipidemia, the literature shows contradictory results for whether or not long-term coffee consumption has a blood pressure-lowering effect [61,62]. Caffeine may increase blood pressure by raising catecholamine levels, leading, in turn, to vasoconstriction [63,64]. However, a recent randomized controlled trial investigating the effect of decaffeinated green coffee bean extract (800 mg per day over eight weeks) in people with MetS found a reduction in systolic blood pressure for the intervention group [65], suggesting that bioactive compounds other than caffeine were involved. It has been postulated that the hypotensive feature of coffee could be attributed to chlorogenic acids through a cortisol-lowering effect [66]. Moreover, coffee is a source of magnesium, potassium, manganese, and niacin [67,68,69], which may all participate in this blood pressure-lowering effect.

Finally, coffee, which is rich in phenolic compounds, is a significant source of dietary antioxidants [22,23,24], and has a higher antioxidant capacity than tea [70,71]. A higher plasma or dietary total antioxidant capacity is associated with lower likelihoods of dyslipidemia, hyperglycemia, and hypertension [72,73,74]. Accordingly, oxidative stress is an important underlying factor that leads to the development of metabolic disorders [75]. The lower antioxidant capacity, as well as the lower prevalence of high-level consumers in our study population, may explain the lack of association that we found between tea consumption and metabolic disorders. Indeed, the beneficial effects of tea consumption on metabolic syndrome reported in epidemiological studies or controlled trials generally appear when the level of consumption is at least 3 cups per day [76,77].

We found no association between coffee consumption and diabetes in our study population of persons with chronic HBV infection. No such association was found in a previous study that we conducted on HCV-infected persons in the same Hepather cohort [78]. Elsewhere, an inverse relationship has been found in the general population [32], and possible mechanisms are highlighted [32,79]. The protective effect of coffee intake on functional beta cell mass [79] may not be strong enough to significantly counteract the compromising effect of HBV infection [80]. Moreover, several clinical trials testing the effects of coffee (or coffee extracts) consumption on glucose metabolism yielded null results [65,81,82,83].

Tobacco use was identified as another behavioral and modifiable risk factor for metabolic disorders in the present study. Current and former tobacco use were both associated with a greater risk of dyslipidemia. This is consistent with data from the general population [84,85,86,87]. This dyslipidemia-inducing effect may be explained by the dysregulation of lipolysis in adipose tissue by nicotine [88]. The lack of any difference between current and former users may be related to a short duration of abstinence (data unavailable for our study). 

Former tobacco use tended to be associated with a higher risk of hypertension, while current use tended to be associated with a lower risk in our models. The hypertensive effect of chronic tobacco use is known [89,90]. One possible explanation for the discrepancy found between former and current users is different exposure in terms of the duration and intensity of smoking, with former smokers possibly being previously long-term heavy smokers (data unavailable). The body weight-lowering effect of tobacco smoking [91] and the weight-gaining effect of quitting tobacco [92] may also explain the opposite tendencies which former and current use had on hypertension [93].

The main strengths of the present study are its large sample size (*n* = 4746) and the presence of demographic, clinical, and socio-behavioral variables. While data for well-known risk factors for metabolic disorders were not available (e.g., diet quality or physical activity), we may expect that adjustment for socio-demographic variables which are generally associated with those risk factors [94,95,96,97,98], at least partially captured such effects. Limitations must also be mentioned. Coffee and tea characteristics (roasting, caffeinated or not, filtered or not, green vs. black, etc.) may influence health effects [76,99,100,101], yet these data were not collected. Further research is needed to check whether the magnitude of the association between coffee or tea intake and metabolic disorders differs according to the type of coffee or tea consumed. Moreover, data on dietary habits such as adding sugar or milk were not collected; however, they may have been partially captured with country of birth, as the latter may reflect culture-driven habits. Using a food frequency questionnaire or a 24-h dietary recall would have improved the robustness of our conclusions. Finally, our study population was limited to people followed in hospital-based specialized services, and socio-behavioral data (including coffee consumption) were only collected during the cohort enrolment visit.

## 5. Conclusions

To conclude, drinking three or more cups of coffee per day was associated with a higher risk of dyslipidemia and a lower risk of hypertension in HBV-infected patients. These two effects are expected to be associated with favorable clinical outcomes. Further studies should explore associated mechanisms, and test whether these seemingly metabolic benefits translate into reduced mortality risks in HBV-infected patients.

This section is not mandatory, but can be added to the manuscript if the discussion is unusually long or complex.

## Figures and Tables

**Table 1:** Study sample characteristics according to coffee consumption (ANRS CO22 Hepather cohort, *n* = 4746).

	Study Population (N = 4746) N (%)	Coffee Consumption N (%)			
**Characteristics** (% of missing)		0 cup/day	1–2 cups/day	≥3 cups/day	*p*-value
**Gender**					
Male	3004 (63.3)	973 (56.6)	1086 (62.0)	945 (74.2)	<10^−3^
Female	1742 (36.7)	746 (43.4)	667 (38.0)	329 (25.8)	
**Age (years)**					
<40	1945 (41.0)	910 (52.9)	643 (36.7)	392 (30.8)	<10^−3^
40–49	1157 (24.4)	384 (22.3)	406 (23.2)	367 (28.8)	
50–59	847 (17.8)	231 (13.4)	317 (18.1)	299 (23.5)	
≥60	797 (16.8)	194 (11.3)	387 (22.1)	216 (17.0)	
**Place of birth**					
France	1377 (29.0)	387 (22.5)	472 (26.9)	518 (40.7)	<10^−3^
Europe ^1^	497 (10.5)	103 (6.0)	208 (11.9)	186 (14.6)	
North Africa	412 (8.7)	83 (4.8)	199 (11.4)	130 (10.2)	
Sub-Saharan Africa ^2^	1753 (36.9)	921 (53.6)	557 (31.8)	275 (21.6)	
Asia	707 (14.9)	225 (13.1)	317 (18.1)	165 (13.0)	
**Body mass index (kg/m^2^) ^3^** (0.9)					
<25 (under or normal weight)	2378 (50.6)	889 (52.2)	881 (50.8)	608 (48.1)	0.259
≥25 and <30 (overweight)	1631 (34.7)	566 (33.2)	601 (34.7)	464 (36.7)	
≥30 (obese)	693 (14.7)	248 (14.6)	252 (14.5)	193 (15.3)	
**Living in a couple** (0.1)					
No	1654 (34.9)	703 (41.0)	563 (32.2)	388 (30.5)	<10^−3^
Yes	3086 (65.1)	1013 (59.0)	1188 (67.8)	885 (69.5)	
**Coffee consumption**					
None	1719 (36.2)	/	/	/	
1–2 cups/day	1753 (36.9)	/	/	/	
≥3 cups/day	1274 (26.8)	/	/	/	
**Tea consumption** (0.3)					
Non-daily	2881 (60.9)	1001 (58.3)	1019 (58.4)	861 (67.8)	<10^−3^
Daily	1851 (39.1)	717 (41.7)	726 (41.6)	408 (32.2)	
**Tea consumption** (0.3)					
<3 cups/day	4240 (89.6)	1521 (88.5)	1586 (90.9)	1133 (89.3)	0.069
≥3 cups/day	492 (10.4)	197 (11.5)	159 (9.1)	136 (10.7)	
**Cannabis use** (0.7)					
Never	4417 (93.7)	1637 (95.8)	1652 (94.9)	1128 (89.3)	<10^−3^
Former	176 (3.7)	38 (2.2)	57 (3.3)	81 (6.4)	
Current	120 (2.5)	34 (2.0)	32 (1.8)	54 (4.3)	
**Tobacco use**					
Never	3072 (64.7)	1369 (79.6)	1140 (65.1)	563 (44.2)	<10^−3^
Former	830 (17.5)	181 (10.5)	338 (19.3)	311 (24.4)	
Current	843 (17.8)	169 (9.8)	274 (15.6)	400 (31.4)	
**Alcohol use** (0.4)					
Abstinent without history of unhealthy use	2711 (57.3)	1149 (67.2)	983 (56.3)	579 (45.5)	<10^−3^
Moderate use	1750 (37.0)	484 (28.3)	664 (38.0)	602 (47.3)	
Current or past unhealthy use	268 (5.7)	77 (4.5)	100 (5.7)	91 (7.2)	
**Living in poverty** (3.0)					
No	2389 (51.9)	707 (42.5)	922 (54.1)	760 (61.4)	<10^−3^
Yes	2214 (48.1)	955 (57.5)	781 (45.9)	478 (38.6)	
**Educational level** (1.4)					
<upper secondary school certificate	2283 (48.8)	803 (47.4)	855 (49.5)	625 (49.8)	0.335
≥upper secondary school certificate	2395 (51.2)	892 (52.6)	872 (50.5)	631 (50.2)	
**Time since HBV diagnosis**—in years (3.3)					
Median [IQR]	9.2 [3.9–17.0]	7.6 [3.2–13.7]	9.5 [4.3–17.7]	11.2 [4.6–19.6]	<10^−3^
**Advanced liver fibrosis ^4^** (11.5)					
No	4020 (95.7)	1450 (95.5)	1494 (94.8)	1076 (97.2)	0.010
Yes	181 (4.3)	68 (4.5)	82 (5.2)	31 (2.8)	
**Dyslipidemia**					
No	4341 (91.5)	1626 (94.6)	1588 (90.6)	1127 (88.5)	<10^−3^
Yes	405 (8.5)	93 (5.4)	165 (9.4)	147 (11.5)	
**Hypertension**					
No	3928 (82.8)	1449 (84.3)	1404 (80.1)	1075 (84.4)	0.001
Yes	818 (17.2)	270 (15.7)	349 (19.9)	199 (15.6)	
**Diabetes**					
No	4390 (92.5)	1604 (93.3)	1602 (91.4)	1184 (92.9)	0.078
Yes	356 (7.5)	115 (6.7)	151 (8.6)	90 (7.1)	

^1^ The category ‘Europe’ included participants from the U.S. (*n* = 2), New Zealand (*n* = 1), and South America (*n* = 11). ^2^ The category ‘Sub-Saharan Africa’ included participants from Haiti (*n* = 44) and the Dominican Republic (*n* = 2). ^3^ World Health Organization categorization [47]. ^4^ Advanced liver fibrosis was defined as an FIB-4 score > 3.25 [43].

**Table 2:** Factors associated with dyslipidemia (ANRS CO22 Hepather cohort, *n* = 4746).

	Univariable Analysis		Multivariable Analysis (Model 1) ^1^ N = 4702		Multivariable Analysis (Model 2) ^1^ N = 4702		Multivariable Analysis (Model 3) ^1^ N = 4590	
	OR [95% CI]	*p*-Value	aOR [95% CI]	*p*-Value	aOR [95% CI]	*p*-Value	aOR [95% CI]	*p*-Value
**Gender**								
Male	1							
Female	0.61 [0.49–0.77]	<10^−3^						
**Age (years)**		**<10^−3^**		**<10^−3^**		**<10^−3^**		**<10^−3^**
<40	1		1		1		1	
40–49	2.93 [2.01–4.27]	<10^−3^	2.51 [1.72–3.69]	<10^−3^	2.51 [1.72–3.69]	<10^−3^	2.24 [1.50–3.34]	<10^−3^
50–59	8.01 [5.65–11.34]	<10^−3^	6.40 [4.47–9.15]	<10^−3^	6.40 [4.47–9.15]	<10^−3^	4.65 [3.13–6.90]	<10^−3^
≥60	9.79 [6.93–13.82]	<10^−3^	7.72 [5.38–11.09]	<10^−3^	7.72 [5.38–11.09]	<10^−3^	4.43 [2.91–6.75]	<10^−3^
**Place of birth**		**<10^−3^**						
France	1							
Europe ^2^	0.93 [0.67–1.28]	0.641						
North Africa	0.70 [0.48–1.02]	0.063						
Sub-Saharan Africa ^3^	0.36 [0.27–0.47]	<10^−3^						
Asia	0.74 [0.55–1.00]	0.050						
**Body mass index (kg/m^2^) ^4^**		**<10^−3^**		**<10^−3^**		**<10^−3^**		
<25 (under or normal weight)	1		1		1			
≥25 and <30 (overweight)	1.76 [1.39–2.22]	<10^−3^	1.48 [1.16–1.89]	0.001	1.48 [1.16–1.89]	0.001		
≥30 (obese)	2.30 [1.74–3.04]	<10^−3^	1.86 [1.39–2.48]	<10^−3^	1.86 [1.39–2.48]	<10^−3^		
**Living in a couple**								
No	1							
Yes	1.40 [1.12–1.76]	0.003						
**Coffee consumption**		**<10^−3^**		**0.033**		**0.033**		**0.009**
None	1		1		1		1	
1–2 cups/day	1.82 [1.40–2.36]	<10^−3^	1.28 [0.97–1.70]	0.083	1.28 [0.97–1.70]	0.083	1.32 [0.99–1.75]	0.061
≥3 cups/day	2.28 [1.74–2.99]	<10^−3^	1.49 [1.10–2.00]	0.009	1.49 [1.10–2.00]	0.009	1.62 [1.19–2.20]	0.002
**Tea consumption**								
Non-daily	1							
Daily	0.80 [0.64–0.99]	0.039						
**Tea consumption**								
<3 cups/day	1							
≥3 cups/day	0.94 [0.67–1.32]	0.720						
**Cannabis use**		**0.222**						
Never	1							
Former	1.29 [0.79–2.11]	0.302						
Current	0.56 [0.25–1.29]	0.173						
**Tobacco use**		**<10^−3^**		**<10^−3^**		**<10^−3^**		**<10^−3^**
Never	1		1		1		1	
Former	2.76 [2.18–3.49]	<10^−3^	1.63 [1.26–2.11]	<10^−3^	1.63 [1.26–2.11]	<10^−3^	1.67 [1.28–2.18]	<10^−3^
Current	1.45 [1.10–1.91]	0.009	1.47 [1.08–1.98]	0.013	1.47 [1.08–1.98]	0.013	1.49 [1.09–2.04]	0.013
**Alcohol use**		**<10^−3^**						
Abstinent without history of unhealthy use	1							
Moderate use	1.59 [1.29–1.97]	<10^−3^						
Current or past unhealthy use	2.12 [1.45–3.10]	<10^−3^						
**Living in poverty**								
No	1							
Yes	0.73 [0.59–0.90]	0.003						
**Educational level**								
<upper secondary school certificate	1							
≥upper secondary school certificate	0.61 [0.49–0.75]	<10^−3^						
**Time since HBV diagnosis (years)**	1.03 [1.03–1.05]	<10^−3^					1.01 [1.00–1.02]	0.011
**Advanced liver fibrosis ^5^**								
No	1		-				-	
Yes	1.14 [0.69–1.88]	0.598	-				-	
**Diabetes**								
No	1		-		-		1	
Yes	4.88 [3.76–6.33]	<10^−3^	-		-		2.61 [1.94–3.52]	<10^−3^
**Hypertension**								
No	1		-		-		1	
Yes	4.22 [3.41–5.23]	<10^−3^	-		-		2.00 [1.55–2.59]	<10^−3^

^1^ In model 1, advanced liver fibrosis, diabetes, and hypertension were not considered eligible for multivariate analyses. In model 2, which was based on model 1, advanced liver fibrosis was considered eligible for multivariate analyses. In model 3, which was also based on model 1, both diabetes and hypertension were considered eligible for multivariate analyses. ^2^ The category ‘Europe’ included participants from the U.S. (*n* = 2), New Zealand (*n* = 1), and South America (*n* = 11). ^3^ The category ‘Sub-Saharan Africa’ included participants from Haiti (*n* = 44) and the Dominican Republic (*n* = 2). ^4^ World Health Organization categorization [47]. ^5^ Advanced liver fibrosis was defined as an FIB-4 score > 3.25 [43]. aOR, adjusted odds ratio; CI, confidence interval; HBV, hepatitis B virus.

**Table 3:** Factors associated with hypertension (ANRS CO22 Hepather cohort, *n* = 4746).

	Univariable Analysis		Multivariable Analysis (Model 1) ^1^ N = 4702		Multivariable Analysis (Model 2) ^1^ N = 4702		Multivariable Analysis (Model 3) ^1^ N = 4590	
	OR [95% CI]	*p*-Value	aOR [95% CI]	*p*-Value	aOR [95% CI]	*p*-Value	aOR [95% CI]	*p*-Value
**Gender**								
Male	1		1		1			
Female	0.75 [0.64–0.89]	0.001	0.78 [0.63–0.95]	0.014	0.79 [0.63–0.97]	0.027		
**Age (years)**		**<10^−3^**		**<10^−3^**		**<10^−3^**		**<10^−3^**
<40	1		1		1		1	
40–49	3.83 [2.84–5.17]	<10^−3^	4.02 [2.93–5.51]	<10^−3^	3.63 [2.60–5.06]	<10^−3^	3.69 [2.71–5.04]	<10^−3^
50–59	11.56 [8.71–15.35]	<10^−3^	13.02 [9.54–17.76]	<10^−3^	12.53 [9.02–17.39]	<10^−3^	10.58 [7.75–14.44]	<10^−3^
≥60	22.62 [17.11–29.91]	<10^−3^	26.58 [19.21–36.78]	<10^−3^	25.03 [17.75–35.30]	<10^−3^	21.62 [15.62–29.90]	<10^−3^
**Place of birth**		**<10^−3^**		**<10^−3^**		**<10^−3^**		**<10^−3^**
France	1		1		1		1	
Europe ^2^	0.71 [0.55–0.93]	0.013	0.77 [0.57–1.05]	0.103	0.74 [0.53–1.03]	0.076	0.79 [0.58–1.07]	0.126
North Africa	0.85 [0.64–1.11]	0.231	0.93 [0.68–1.28]	0.656	0.95 [0.68–1.31]	0.740	0.85 [0.61–1.19]	0.341
Sub-Saharan Africa ^3^	0.57 [0.47–0.68]	<10^−3^	1.54 [1.20–1.98]	0.001	1.55 [1.19–2.01]	0.001	1.55 [1.20–1.99]	0.001
Asia	0.54 [0.42–0.70]	<10^−3^	0.97 [0.73–1.30]	0.861	1.02 [0.75–1.39]	0.879	0.90 [0.68–1.21]	0.491
**Body mass index (kg/m^2^) ^4^**		**<10^−3^**		**<10^−3^**		**<10^−3^**		**<10^−3^**
<25 (under or normal weight)	1		1		1		1	
≥25 and <30 (overweight)	1.82 [1.52–2.17]	<10^−3^	1.56 [1.271.90]	<10^−3^	1.58 [1.27–1.95]	<10^−3^	1.44 [1.18–1.76]	<10^−3^
≥30 (obese)	4.24 [3.47–5.19]	<10^−3^	3.97 [3.12–5.04]	<10^−3^	4.31 [3.36–5.55]	<10^−3^	3.25 [2.55–4.14]	<10^−3^
**Living in a couple**								
No	1							
Yes	1.29 [1.10–1.52]	0.002						
**Coffee consumption**		**0.001**		**0.001**		**0.004**		**0.003**
None	1		1		1		1	
1–2 cups/day	1.33 [1.12–1.59]	0.001	0.90 [0.73–1.11]	0.338	0.91 [0.72–1.13]	0.387	0.92 [0.74–1.14]	0.425
≥3 cups/day	0.99 [0.81–1.21]	0.949	0.64 [0.50–0.82]	0.001	0.65 [0.50–0.85]	0.001	0.66 [0.51–0.85]	0.001
**Tea consumption**								
Non-daily	1							
Daily	0.77 [0.66–0.90]	0.001						
**Tea consumption**								
<3 cups/day	1							
≥3 cups/day	0.88 [0.68–1.14]	0.329						
**Cannabis use**		**0.001**						
Never	1							
Former	0.59 [0.37–0.95]	0.028						
Current	0.33 [0.16–0.68]	0.002						
**Tobacco use**		**<10^−3^**		**0.013**		**0.018**		**0.007**
Never	1		1		1		1	
Former	2.20 [1.84–2.63]	<10^−3^	1.19 [0.95–1.48]	0.125	1.21 [0.96–1.52]	0.111	1.26 [1.01–1.56]	0.038
Current	0.63 [0.49–0.80]	<10^−3^	0.75 [0.57–1.00]	0.047	0.76 [0.56–1.02]	0.070	0.78 [0.59–1.03]	0.083
**Alcohol use**		**<10^−3^**						
Abstinent without history of unhealthy use	1							
Moderate use	1.26 [1.07–1.48]	0.005						
Current or past unhealthy use	2.42 [1.83–3.21]	<10^−3^						
**Living in poverty**								
No	1							
Yes	0.92 [0.78–1.07]	0.258						
**Educational level**								
<upper secondary school certificate	1		1		1			
≥upper secondary school certificate	0.56 [0.48–0.65]	<10^−3^	0.81 [0.67–0.96]	0.017	0.81 [0.67–0.98]	0.031		
**Time since HBV diagnosis—(years)**								
**Advanced liver fibrosis ^5^**	1.03 [1.02–1.04]	<10^−3^						
No	1				1			
Yes	3.45 [2.54–4.70]	<10^−3^	-		1.50 [1.05–2.13]	0.024	-	
**Diabetes**			-				-	
No	1		-		-		1	
Yes	7.23 [5.77–9.05]	<10^−3^	-		-		2.93 [2.23–3.84]	<10^−3^
**Hypertension**								
No	1		-		-		1	
Yes	4.22 [3.40–5.23]	<10^−3^	-		-		1.98 [1.52–2.57]	<10^−3^

^1^ In model 1, advanced liver fibrosis, diabetes, and hypertension were not considered eligible for multivariate analyses. Model 2 is based on model 1, but advanced liver fibrosis is considered eligible for multivariate analyses. Model 3 is based on model 1, but both diabetes and hypertension were considered eligible for multivariate analyses. ^2^ The category ‘Europe’ included participants from the U.S. (*n* = 2), New Zealand (*n* = 1), and South America (*n* = 11). ^3^ The category ‘Sub-Saharan Africa’ included participants from Haiti (*n* = 44) and the Dominican Republic (*n* = 2). ^4^ World Health Organization categorization [47]. ^5^ Advanced liver fibrosis was defined as an FIB-4 score > 3.25 [43]. aOR, adjusted odds ratio; CI, confidence interval; HBV, hepatitis B virus.

**Table 4:** Factors associated with diabetes (ANRS CO22 Hepather cohort, *n* = 4746).

	Univariable Analysis		Multivariable Analysis (Model 1) ^1^ N = 4702		Multivariable Analysis (Model 2) ^1^ N = 4702		Multivariable Analysis (Model 3) ^1^ N = 4590	
	OR [95% CI]	*p*- Value	aOR [95% CI]	*p*-Value	aOR [95% CI]	*p*-Value	aOR [95% CI]	*p*-Value
**Gender**								
Male	1		1					
Female	0.75 [0.59–0.94]	0.014	0.75 [0.58–0.98]	0.032				
**Age (years)**		**<10^−3^**		**<10^−3^**		**<10^−3^**		**<10^−3^**
<40	1		1		1		1	
40–49	2.74 [1.82–4.15]	<10^−3^	2.60 [1.68–4.02]	<10^−3^	2.59 [1.62–4.14]	<10^−3^	2.15 [1.38–3.35]	0.001
50–59	7.49 [5.13–10.94]	<10^−3^	7.86 [5.22–11.83]	<10^−3^	7.79 [5.07–11.99]	<10^−3^	4.81 [3.10–7.44]	<10^−3^
≥60	11.44 [7.93–16.53]	<10^−3^	12.73 [8.34–19.43]	<10^−3^	11.71 [7.57–18.12]	<10^−3^	6.41 [4.04–10.17]	<10^−3^
**Place of birth**		**<10^−3^**		**0.048**				**0.016**
France	1		1				1	
Europe ^2^	1.14 [0.78–1.64]	0.501	1.20 [0.80–1.80]	0.387			1.25 [0.82–1.92]	0.299
North Africa	1.85 [1.31–2.61]	0.001	1.67 [1.11–2.50]	0.014			1.91 [1.27–2.87]	0.002
Sub-Saharan Africa ^3^	0.74 [0.56–0.98]	0.037	1.39 [0.99–1.95]	0.056			1.42 [1.00–2.02]	0.051
Asia	0.91 [0.64–1.29]	0.607	1.64 [1.11–2.42]	0.013			1.67 [1.11–2.50]	0.013
**Body mass index (kg/m^2^) ^4^**		**<10^−3^**		**<10^−3^**		**<10^−3^**		**<10^−3^**
<25 (under or normal weight)	1		1		1		1	
≥25 and <30 (overweight)	2.87 [2.17–3.79]	<10^−3^	2.41 [1.78–3.25]	<10^−3^	2.14 [1.57–2.92]	<10^−3^	2.23 [1.64–3.04]	<10^−3^
≥30 (obese)	6.06 [4.51–8.14]	<10^−3^	5.13 [3.67–7.17]	<10^−3^	5.02 [3.61–6.99]	<10^−3^	3.73 [2.63–5.27]	<10^−3^
**Living in a couple**								
No	1							
Yes	0.94 [0.75–1.18]	0.581						
**Coffee consumption**		**0.079**						
None	1							
1–2 cups/day	1.31 [1.02–1.69]	0.034						
≥ 3 cups/day	1.06 [0.80–1.41]	0.689						
**Tea consumption**								
Non-daily	1							
Daily	0.83 [0.67–1.05]	0.117						
**Tea consumption**								
<3 cups/day	1							
≥3 cups/day	0.72 [0.49–1.07]	0.109						
**Cannabis use**		**0.080**						
Never	1							
Former	0.71 [0.37–1.37]	0.311						
Current	0.30 [0.10–0.96]	0.043						
**Tobacco use**		**<10^−3^**						
Never	1							
Former	1.79 [1.39–2.31]	<10^−3^						
Current	0.82 [0.59–1.13]	0.225						
**Alcohol use**		**<10^−3^**		**0.024**				**0.011**
Abstinent without history of unhealthy use	1		1				1	
Moderate use	0.80 [0.63–1.02]	0.076	0.75 [0.57–0.99]	0.041			0.73 [0.55–0.97]	0.030
Current or past unhealthy use	2.21 [1.54–3.17]	<10^−3^	1.29 [0.84–1.96]	0.240			1.37 [0.89–2.10]	0.152
**Living in poverty**								
No	1		1		1		1	
Yes	1.32 [1.06–1.65]	0.013	1.52 [1.17–1.98]	0.002	1.73 [1.33–2.25]	<10^−3^	1.44 [1.10–1.88]	0.008
**Educational level**								
<upper secondary school certificate	1							
≥upper secondary school certificate	0.53 [0.42–0.66]	<10^−3^						
**Time since HBV diagnosis (years)**	1.00 [0.99–1.01]	0.989						
**Advanced liver fibrosis ^5^**								
No	1		-		1		-	
Yes	3.60 [2.48–5.23]	<10^−3^	-		2.14 [1.40–3.28]	<10^−3^	-	
**Diabetes**								
No	1		-		-		1	
Yes	7.23 [5.77–9.05]	<10^−3^	-		-		2.99 [2.28–3.92]	<10^−3^
**Hypertension**								
No	1		-		-		1	
Yes	4.88 [3.76–6.33]	<10^−3^	-		-		2.49 [1.84–3.38]	<10^−3^

^1^ In model 1, advanced liver fibrosis, diabetes, and hypertension were not considered eligible for multivariate analyses. Model 2 is based on model 1, but advanced liver fibrosis is considered eligible for multivariate analyses. Model 3 is based on model 1, but both diabetes and hypertension were considered eligible for multivariate analyses. ^2^ The category ‘Europe’ included participants from the U.S. (*n* = 2), New Zealand (*n* = 1), and South America (*n* = 11). ^3^ The category ‘Sub-Saharan Africa’ included participants from Haiti (*n* = 44) and the Dominican Republic (*n* = 2). ^4^ World Health Organization categorization [47]. ^5^ Advanced liver fibrosis was defined as an FIB-4 score > 3.25 [43]. aOR, adjusted odds ratio; CI, confidence interval; HBV, hepatitis B virus.

## Data Availability

Not applicable.

## Supplementary Materials

The following supporting information can be downloaded at: https://www.mdpi.com/article/10.3390/antiox11020379/s1, Table S1: Characteristics of included and excluded participants (ANRS CO22 Hepather cohort).
