# Supplementary material for: Metabolic Disorders in Patients with Chronic Hepatitis B Virus Infection: Coffee as a Panacea? (ANRS CO22 Hepather Cohort)
Source: Antioxidants (Basel). 2022 Feb 14;11(2):379. doi: 10.3390/antiox11020379 (PMC8869416; doi:10.3390/antiox11020379)
Supplement: Supplementary file 1 [file antioxidants-11-00379-s001.zip › antioxidants-1579039-Supplementary.pdf]

Supplementary Table 1: Characteristics of included and excluded participants (ANRS CO22 Hepather cohort)

Characteristics (% of missing)	Excluded (N = 467)	Included (N = 4746)	p-value
**Gender**			
Male	297 (63.6)	3004 (63.3)	0.897
Female	170 (36.4)	1742 (36.7)	
**Age (years)**			
< 40	156 (33.4)	1945 (41.0)	$< 10^{-3}$
40-49	127 (27.2)	1157 (24.4)	
50-59	115 (24.6)	847 (17.8)	
≥ 60	69 (14.8)	797 (16.8)	
**Place of birth**			
France	99 (21.6)	1377 (29.0)	$< 10^{-3}$
Europe[1]	81 (17.6)	497 (10.5)	
North Africa	25 (5.4)	412 (8.7)	
Sub-Saharan Africa[2]	187 (40.7)	1753 (36.9)	
Asia	67 (14.6)	707 (14.9)	
**Body mass index (kg/m^2)**[3] (0.9)			
<25 (under or normal weight)	238 (55.2)	2378 (50.6)	0.181
≥25 and <30 (overweight)	135 (31.3)	1631 (34.7)	
≥30 (obese)	58 (13.5)	693 (14.7)	
**Living in a couple** (0.1)			
No	168 (38.9)	1654 (34.9)	0.096
Yes	264 (61.1)	3086 (65.1)	
**Coffee consumption**			
None	130 (39.5)	1719 (36.2)	0.108
1-2 cups/day	128 (38.9)	1753 (36.9)	
≥ 3 cups/day	71 (21.6)	1274 (26.8)	
**Tea consumption** (0.3)			
Non-daily	171 (52.0)	2881 (60.9)	0.001
Daily	158 (48.0)	1851 (39.1)	
**Tea consumption** (0.3)			
< 3 cups/day	286 (86.9)	4240 (89.6)	0.127
≥ 3 cups/day	43 (13.1)	492 (10.4)	
**Cannabis use** (0.7)			
Never	391 (87.7)	4417 (93.7)	$< 10^{-3}$
Former	29 (6.5)	176 (3.7)	

Current	26 (5.8)	120 (2.5)	
**Tobacco use**			
Never	275 (60.8)	3072 (64.7)	< 10^{-3}
Former	62 (13.7)	830 (17.5)	
Current	115 (25.4)	843 (17.8)	
**Alcohol use** (0.4)			
Abstinent without history of unhealthy use	261 (58.4)	2711 (57.3)	< 10^{-3}
Moderate use	132 (29.5)	1750 (37.0)	
Current or past unhealthy use	54 (12.1)	268 (5.7)	
**Living in poverty** (3.0)			
No	150 (40.3)	2389 (51.9)	< 10^{-3}
Yes	222 (59.7)	2214 (48.1)	
**Educational level** (1.4)			
< upper secondary school certificate	218 (54.8)	2283 (48.8)	0.022
≥ upper secondary school certificate	180 (45.2)	2395 (51.2)	
**Time since HBV diagnosis** – *in years* (3.3)			
Median [IQR]	10.3 [4.7 – 17.4]	9.2 [3.9 – 17.0]	0.011
**Advanced liver fibrosis**[4] (11.5)			
No	342 (83.4)	4020 (95.7)	< 10^{-3}
Yes	68 (16.6)	181 (4.3)	
**Dyslipidemia**			
No	427 (94.1)	4341 (91.5)	0.056
Yes	27 (5.9)	405 (8.5)	
**Hypertension**			
No	352 (77.4)	3928 (82.8)	0.004
Yes	103 (22.6)	818 (17.2)	
**Diabetes**			
No	425 (93.0)	4390 (92.5)	0.698
Yes	32 (7.0)	356 (7.5)	

[1]The category 'Europe' included participants from the U.S. (*n* = 2), New Zealand (*n* = 1), and South America (*n* = 11).

[2]The category 'Sub-Saharan Africa' included participants from Haiti (*n* = 44) and the Dominican Republic (*n* = 2).

[3]World Health Organization categorization (47).

[4]Advanced liver fibrosis was defined as an FIB-4 score > 3.25 (43).

HBV, hepatitis B virus; IQR, interquartile range.
